# Firearm-Related Injury and Death: A U.S. Health Care Crisis in Need of Health Care Professionals

**DOI:** 10.1371/journal.pmed.1002430

**Published:** 2017-10-09

**Authors:** Darren B. Taichman, Howard Bauchner, Jeffrey M. Drazen, Christine Laine, Larry Peiperl

**Affiliations:** 1 Executive Deputy Editor, Annals of Internal Medicine; 2 Editor-in-Chief, JAMA (Journal of the American Medical Association) and the JAMA Network; 3 Editor-in-Chief, New England Journal of Medicine; 4 Editor-in-Chief, Annals of Internal Medicine; 5 Chief Editor, PLOS Medicine

## Abstract

The U.S.-based Editors of ICMJE journals call for health-care professionals to act against the public health crisis of injury and death from guns.

What would happen if on one day more than 50 people died and over 10 times that many were harmed by an infectious disease in the United States? Likely, our nation’s esteemed and highly capable public health infrastructure would gear up to care for those harmed and study the problem. There would be a rush to identify the cause, develop interventions, and refine them continually until the threat is eliminated or at least contained. In light of the risks to public health (after all, over 500 people have been harmed already!), health care professionals would sound the alarm. We would demand funding. We would go to conferences to learn what is known and what we should do. We would form committees at our institutions to plan local responses to protect our communities. The United States would spend millions or more in short order to assure public safety, and no elected officials would conceive of getting in the way. Rather, they would compete to be calling the loudest for the funds and focus required to protect our people. Americans should be proud of our prowess at and commitment to addressing public health crises.

Yet, here we are again with another editorial about the public health crisis of firearm-related injury and death following what used to be unthinkable, this time a mass murder and casualties at a concert in Las Vegas. We’ve written it all before. The staggering numbers killed annually. The numbers left permanently disabled. The families left to cope with the loss of loved ones or to care for those broken but not killed by a bullet. As health care professionals, we seem powerless. This public health crisis seems beyond the reach of our tools.

Is there really nothing health care professionals can do? We think there is a lot. We need to each ask ourselves what we have done to apply our knowledge and skills to help address the problem since the moment of silence that followed the last mass shooting. More silence is not the answer. Have we demanded funding to adequately study the problem and test solutions? Have we participated in such studies? Have we mobilized forces at our institutions to plan strategies to lower the risks in our communities? Have we talked to our patients about gun safety and effectively challenged policies that would enforce our silence on this matter? Some of our colleagues have. We should be proud of them, but they need all of our help. And so do our patients.

Here’s a short list of how health care professionals can use our skills and voices to fight the threat that firearms present to health in the United States.

Educate yourself. Read the background materials and proposals for sensible firearm legislation from health care professional organizations. Make a phone call and write a letter to your local, state, and federal legislators to tell them how you feel about gun control. Now. Don’t wait. And do it again at regular intervals. Attend public meetings with these officials and speak up loudly as a health care professional. Demand answers, commitments, and follow-up. Go to rallies. Join, volunteer for, or donate to organizations fighting for sensible firearm legislation. Ask candidates for public office where they stand and vote for those with stances that mitigate firearm-related injury.

Meet with the leaders at your own institutions to discuss how to leverage your organization’s influence with local, state, and federal governments. Don’t let concerns for perceived political consequences get in the way of advocating for the well-being of your patients and the public. Let your community know where your institution stands and what you are doing. Tell the press.

Educate yourself about gun safety. Ask your patients if there are guns at home. How are they stored? Are there children or others at risk for harming themselves or others? Direct them to resources to decrease the risk for firearm injury, just as you already do for other health risks. Ask if your patients believe having guns at home makes them safer, despite evidence that they increase the risk for homicide, suicide, and accidents.

Don’t be silent. We don’t need more moments of silence to honor the memory of those who have been killed. We need to honor their memory by preventing a need for such moments. As health care professionals, we don’t throw up our hands in defeat because a disease seems to be incurable. We work to incrementally and continuously reduce its burden. That’s our job.

Will yet another commentary about the ravages of firearm-related harm change anything? Probably not—our journals have published far too many following prior firearm-enabled catastrophes. The only thing that will change the world for the better is a group of people who believe that they can change the world. With regard to firearm-related injury and death, let’s each be part of that group.

